# Genomic Deletion of PfHRP2 and PfHRP3 in *Plasmodium falciparum* Strains, Ethiopia, 2009

**DOI:** 10.3201/eid3107.241676

**Published:** 2025-07

**Authors:** Tamirat Gebru Woldearegai, Tina Krüger, Sindew Mekasha Feleke, Hassen Mamo, Tesfaye Gelanew, Vanessa Krohmer, Sabine Belard, Peter G. Kremsner, Jana Held, Miriam Rodi, Andrea Kreidenweiss

**Affiliations:** Institute of Tropical Medicine, University Hospital Tübingen, Tübingen, Germany (T.G. Woldearegai, T. Krüger, V. Krohmer, S. Belard, P.G. Kremsner, J. Held, M. Rodi, A. Kreidenweiss); German Center for Infection Research, partner site Tübingen, Tübingen (T.G. Woldearegai, S. Belard, P.G. Kremsner, J. Held, A. Kreidenweiss); La Trobe University, Melbourne, Victoria, Australia (S.M. Feleke); Ethiopian Public Health Institute, Addis Ababa, Ethiopia (S.M. Feleke); College of Natural and Computational Sciences, Addis Ababa University, Addis Ababa (S.M. Feleke, H. Mamo); Armauer Hansen Research Institute, Addis Ababa (T. Gelanew); Centre de Recherches Médicales de Lambaréné, Lambaréné, Gabon (P.G. Kremsner, J. Held, A. Kreidenweiss)

**Keywords:** malaria, parasites, Plasmodium falciparum, gene deletion, vector-borne infections, Ethiopia

## Abstract

*Plasmodium falciparum* strains lacking *P. falciparum* histidine-rich protein 2 (PfHRP2) and PfHRP3 threaten malaria rapid test reliability. We show that *pfhrp2/pfhrp3–*deleted parasites circulated in Ethiopia as early as 2009, before widespread PfHRP2-based rapid test use, and had high *pfhrp3* deletion prevalence. Monitoring of *pfhrp2* and of *pfhrp3* deletions is needed.

Malaria caused by *Plasmodium falciparum* remains a major health problem. In 2023, an estimated 263 million cases and 597,000 deaths were seen worldwide; most were in Africa. Introduction of rapid diagnostic tests (RDTs) has substantially increased malaria diagnosis and malaria control. *P. falciparum* histidine-rich protein 2 (PfHRP2)–detecting RDTs rely on monoclonal antibodies raised against PfHRP2. Those monoclonal antibodies cross-detect PfHRP3 because of shared amino acid repeats.

The development of malaria RDTs began in the early 1990s; however, it was not until 2008 that quality-controlled and reliable RDTs became available. The World Health Organization policy shift in 2010 to a test-and-treat strategy boosted widespread use of RDTs, and RDT sales increased to >415 million in 2022 (*1*). In 2010 in the Amazon region of Peru, researchers identified the first *P. falciparum* strains that lacked the *pfhrp2* gene (and *pfhrp3* gene) and caused false-negative PfHRP2 RDT results (*2*). Subsequent studies identified *pfhrp2* gene*–*deleted and *pfhrp3* gene*–*deleted parasites in other malaria-endemic regions; the highest frequencies were reported in the Amazon region in South America and parts of East Africa, including Ethiopia (*3*). The high frequency of gene deletions in countries in East Africa has already led to a policy switch toward non-PfHRP2 RDTs in Eritrea, Djibouti, and, in 2022, Ethiopia (*4*), despite the lack of reliable alternative RDT types.

We suspected that treatment guided by PfHRP2-based RDTs selects for PfHRP2 test–negative parasites that can be further transmitted and spread (*3*). In the Amazon region of Peru, where the first *pfhrp2*-deleted and *pfhrp3-*deleted parasites were found, PfHRP2-based RDTs were not in common use (*2*). To shed light on the multifactorial forces driving the spread of parasites with gene deletions, we retrospectively analyzed samples collected in Ethiopia in 2009, which was a time when PfHRP2-based RDTs were not yet used globally. We obtained the samples from eastern and southwestern regions of the country (Appendix Figure 1).

We used 4-plex quantitative PCR (qPCR) analysis on 89 samples that had already tested positive for *P. falciparum* monoinfection by using species-specific PCR as previously reported (*5*) and that were available in sufficient quantities for testing (*6*) (Appendix); 74 were quality confirmed. Of those 74 samples, 2 (2.7%) were negative for *pfhrp2* and *pfhrp3* (*pfhrp2*^−^/*pfhrp3*^−^) (Figure). We did not detect any *pfhrp2* single deletions (*pfhrp2*^−^/*pfhrp3^+^*), but 21 samples (28.4%) lacked *pfhrp3* (*pfhrp2^+^*/*pfhrp3*^−^). Because sample size was limited, we were unable to analyze regional differences. Most (63/74) samples were from the southwestern (Jimma) region, where >90% of the deletions were found (Table). PfHRP2 RDTs were not performed on the sample set.

**Figure:**
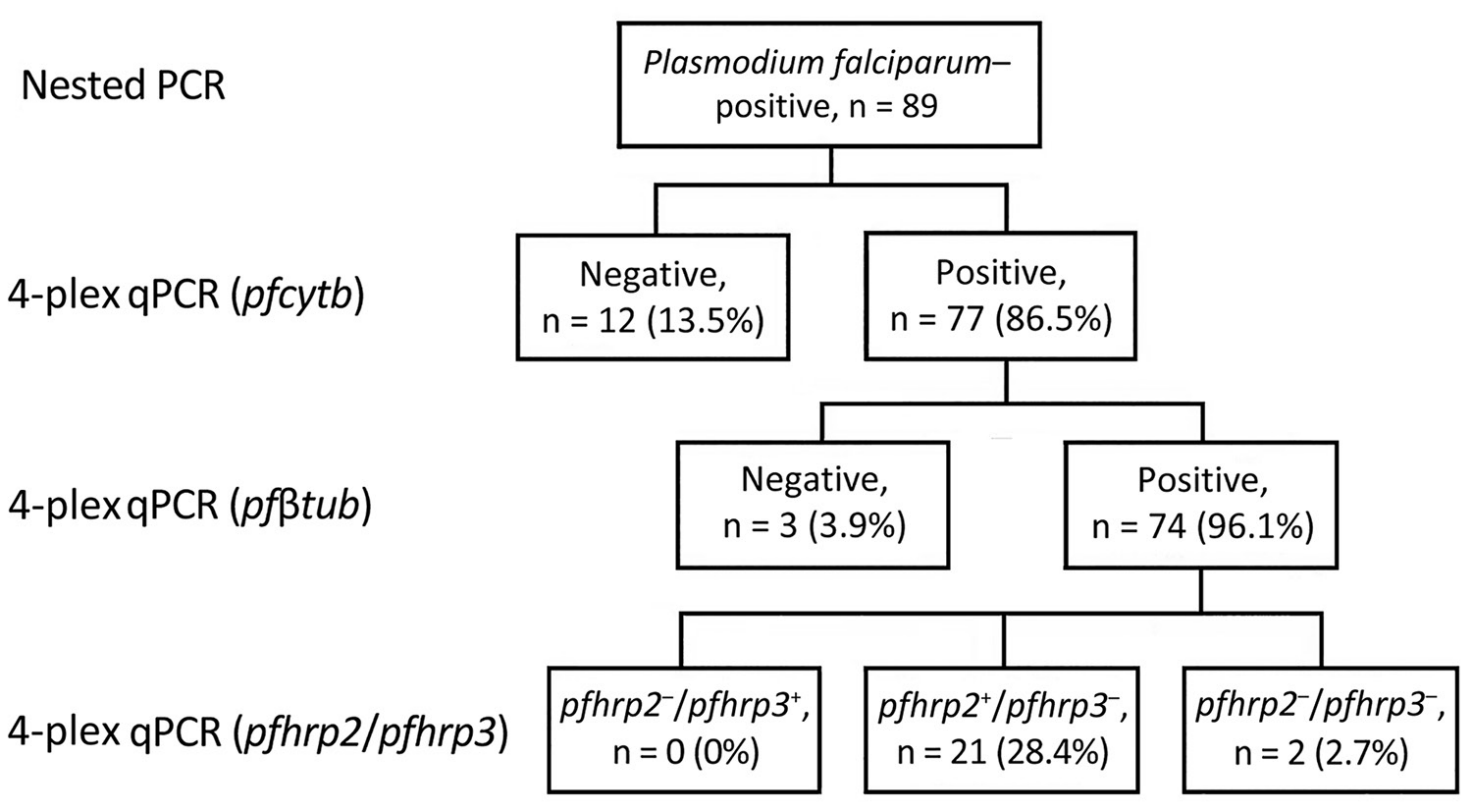
*pfhrp2* and *pfhrp3* deletion frequency in genomic deletion of PfHRP2 and PfHRP3 antigens in *Plasmodium falciparum* strains, Ethiopia, 2009**.** *P. falciparum–*positive samples from 89 persons, previously identified by species-specific PCR were analyzed by 4-plex qPCR for the presence of *P. falciparum* by *pfcytb* to confirm DNA quality and quantity by amplification of the single copy gene *pfβtub* and then for deletion of *pfhrp2* and *pfhrp3*. *pfcytb, P. falciparum* cytochrome b; *pfβtub, P. falciparum* β-tubulin; *pfhrp*, *P. falciparum* histidine-rich protein; qPCR, quantitative PCR.

**Table:** Study population and *pfhrp2* and *pfhrp3* deletion outcomes per study region in genomic deletion of PfHRP2 and PfHRP3 in *Plasmodium falciparum* strains, Ethiopia, 2009*

Study sites	No. samples analyzed	Patient median age, y (range)	Patient sex, %		*pfcytb*^+^/*pfβtub*^+^, no.	*pfhrp2^+^*/*pfhrp3*^−^*,* no. (%)†	*pfhrp2*^−^/*pfhrp3^+^*, no. (%)†	*pfhrp2*^−^/*pfhrp3*^−^, no. (%)†
			F	M				
Jimma	66	18 (1–73)	46	54	63	19 (30.2)	0 (0)	2 (3.2)
Harar	23	25 (6–60)	30	70	11	2 (18.2)	0 (0)	0 (0)
Total	89	21 (1–73)	38	62	74	21 (28.4)	0 (0)	2 (2.7)

*pfβtub, P. falciparum β-tubulin; *pfcytb: P. falciparum* cytochrome b; PfHRP, *P. falciparum* histidine-rich protein. †Percentages were calculated by using the total number of eligible samples (*pfcytb*^+^/*pfbtub*^+^) from each site as denominator.

To further profile the *pfhrp3* gene locus in the 23 samples lacking *pfhrp3* (targeting the 5′ end of *pfhrp3* exon 2), we reassessed the samples by using a modified 4-plex qPCR that targets the 3′ region of *pfhrp3* exon 2 (*7*) (Appendix Figure 2). Of 21 analyzed samples (2 samples had insufficient material), 7 samples were positive for *pfhrp3* (Appendix Table 1). We profiled 5 of those samples (2 samples lacked material) by PCR (primer pairs 3, 4, and 5) that spanned various regions within the *pfhrp3* locus. All 5 samples had a *pfhrp3* gene deletion (Appendix Table 2, Figure 2). We therefore recommend continued use of unmodified 4-plex qPCR (*6*).

Studies of *pfhrp2* and *pfhrp3* deletions in the *P. falciparum* population in Africa with a sufficiently large sample size are lacking (*8*). Before intensive use of PfHRP2-based RDTs, parasites with *pfhrp2* deletions were already present but at very low frequencies and only in association with *pfhrp3* deletions. In contrast, the percentage (28%) of *pfhrp3*-deleted parasites was surprisingly high and agrees with multiple studies from Ethiopia conducted since 2015 (Appendix Table 3).

Our data clarify the emergence and spread of PfHRP2 diagnostic–resistant parasites, supporting Feleke et al. (*3*). Frequently occurring *pfhrp3* deletions might favor selection and spread of occasionally occurring *pfhrp2* deletions under the selective pressure of intensive use of PfHRP2 RDTs followed by antimalarial treatment. Studies published in 2020 and 2021 identified a major role of PfHRP3 in the accuracy of PfHRP2 RDTs, particularly at low parasitemia, where cross-binding can mask *pfhrp2* deletions and result in a positive test (*9*,*10*). In contrast, absence of PfHRP3 in *pfhrp2*-deleted strains results in a false-negative RDT and ultimately leads to positive selection of *pfhrp2*-deleted *P. falciparum*. Those results are particularly relevant in areas of low transmission and with extensive use of PfHRP2 RDTs and antimalarial treatment (*3*; O.J. Watson et al., unpub. data, http://medrxiv.org/lookup/doi/10.1101/2023.10.21.23297352). The frequency of *pfhrp2* and *pfhrp3* deletions is much lower in West and Central Africa countries that have a high transmission rate (O.J. Watson et al., unpub. data).

Use of different molecular tests provided valuable insights into the challenges of deletion detection and nature of *pfhrp3* gene deletion. We confirmed 4-plex qPCR results by using 3 PCRs with commonly used primers and highlight that outcomes might vary depending on the assays applied. pfhrp3 deletions might contribute to the spread of pfhrp2-deleted *P. falciparum* and should be routinely monitored along with pfhrp2 in deletion surveillance studies.

Appendix Genomic deletion of PfHRP2 and PfHRP3 antigens in *Plasmodium falciparum* strains, Ethiopia, 2009.
