# Supplementary material for: Genomic Deletion of PfHRP2 and PfHRP3 in Plasmodium falciparum Strains, Ethiopia, 2009
Source: Emerg Infect Dis. 2025 Jul;31(7):1466–8. doi: 10.3201/eid3107.241676 (PMC12205445; doi:10.3201/eid3107.241676)
Supplement: Appendix — Genomic deletion of PfHRP2 and PfHRP3 antigens in Plasmodium falciparum strains, Ethiopia, 2009. [file 24-1676-Techapp-s1.pdf]

Article DOI: https://doi.org/10.3201/eid3107.241676

EID cannot ensure accessibility for Supplemental Materials supplied by authors. Readers who have difficulty accessing supplementary content should contact the authors for assistance.

Genomic Deletion of PfHRP2 and PfHRP3 Antigens in *Plasmodium falciparum* Strains, Ethiopia, 2009

Appendix

Material and Methods

Study Design, Population, and Sample Collection

This study is a retrospective analysis of 89 stored samples that have been collected from October to November 2009 in a cross-sectional study aiming to molecular characterize Plasmodium parasites in Ethiopian persons. The study design and outcomes have been published previously and were approved by the Ethical Clearance Committee of Haramaya University-College of Health and Medical Sciences, the Harari and Oromia Regional State Health Bureau, and additionally by the National Health Research Ethics Review Committee (NERC) (ref. no. RDHE/28–90/2002) in Ethiopia (*1*). Briefly, blood samples were collected from 1,931 febrile persons of all ages presenting to health centers during the study period in the study areas. *Plasmodium vivax* and *P. falciparum* infections were diagnosed by thick blood smear microscopy. For molecular *Plasmodium* detection and species identification, DNA was extracted by using the QIAamp DNA Mini and Blood Mini kit (QIAGEN), and a species-specific nested PCR assay was performed. Of the 1,931 persons screened, 205 were positive for *P. falciparum* and/or *P. vivax* by species-specific PCR as previously reported (1). Of 94 persons with *P. falciparum* mono-infection, sufficient template DNA (stored at −20°C) was available for 89 persons and was subjected to *pfhrp2*/*pfhrp3* deletion analysis.

Molecular *pfhrp2* and *pfhrp3* Detection

To assess the status of *pfhrp2* and *pfhrp3* genes, the established hydrolysis probe-based, quantitative real-time PCR (4plex qPCR) was performed on the 89 samples that were previously

PCR positive for *P. falciparum*. In every run, the *P. falciparum* laboratory strain 3D7 was included as positive control for both *pfhrp2* and *pfhrp3*, whereas strains Dd2 and HB3 were used as negative controls for absence of *pfhrp2* or *pfhrp3*, respectively. The 4plex qPCR (*2*) assay simultaneously detects *P. falciparum* by cytochrome b gene (*pfcytb*) amplification, confirms template DNA quality and amount for single copy gene amplification by amplifying single copy gene β-tubulin (*pfbtub*), and detects *pfhrp2* and *pfhrp3* single-copy genes if present. Oligo sequences and assay conditions were applied as described previously (*3*). In the modified 4plex qPCR, the *pfhrp3* assay was replaced by *pfhrp3* oligonucleotide sequences targeting the 3′ end of *pfhrp3* exon 2 (*4*). Assays were read using the LightCycler 480 II (Roche Diagnostics, Basel, Switzerland). Threshold for positivity was set to Cq ≤35 per respective target gene. A detailed description of the 4plex qPCR primer/probe sequences and assay conditions is presented in Appendix Tables 4 and 5, respectively.

pfhrp3 Gene Profiling

pfhrp3 gene locus was profiled by 3 additional PCRs (Appendix Tables 4, 5). All amplicons were analyzed by using automated capillary gel electrophoresis (QIAxcel, QIAGEN).

Data Analysis

Descriptive data analysis was conducted by using the R statistical software package and SPSS to examine background data and the patterns of *pfhrp2*/*pfhrp3* deletions. The frequencies of single and double *pfhrp2*/*pfhrp3* deletions were compared across various demographic variables, including age groups, sex, and study sites to assess whether specific populations or locations were more affected by those genetic deletions. A geographic information system software was used to generate the study area map.

References

1. Woldearegai TG, Kremsner PG, Kun JFJ, Mordmüller B. *Plasmodium vivax* malaria in Duffy-negative individuals from Ethiopia. Trans R Soc Trop Med Hyg. 2013;107:328–31. PubMed https://doi.org/10.1093/trstmh/trt016

2. Kreidenweiss A, Trauner F, Rodi M, Koehne E, Held J, Wyndorps L, et al. Monitoring the threatened utility of malaria rapid diagnostic tests by novel high-throughput detection of *Plasmodium falciparum* hrp2 and hrp3 deletions: a cross-sectional, diagnostic accuracy study. EBioMedicine. 2019;50:14–22. PubMed https://doi.org/10.1016/j.ebiom.2019.10.048

3. Krueger T, Ikegbunam M, Lissom A, Sandri TL, Ntabi JDM, Djontu JC, et al. Low prevalence of *Plasmodium falciparum* histidine-rich protein 2 and 3 gene deletions—a multiregional study in Central and West Africa. Pathogens. 2023;12:455. PubMed https://doi.org/10.3390/pathogens12030455

4. Grignard L, Nolder D, Sepulveda N, Berhane A, Mihreteab S, Kaaya R, et al. Corrigendum to 'A novel multiplex qPCR assay for detection of *Plasmodium falciparum* with histidine-rich protein 2 and 3 (pfhrp2 and pfhrp3) deletions in polyclonal infections'. EBioMedicine. 2021;65:103261. PubMed https://doi.org/10.1016/j.ebiom.2021.103261

5. Kamaliddin C, Burke-Gaffney J, Ashraf S, Castañeda-Mogollón D, Adamu A, Mekonen Tefa B, et al. A Countrywide survey of hrp2/3 deletions and kelch13 mutations co-occurrence in Ethiopia. J Infect Dis. 2024;230:e1394–401. PubMed https://doi.org/10.1093/infdis/jiae373

6. Mekonen B, Dugassa S, Feleke SM, Dufera B, Gidisa B, Adamu A, et al. Widespread pfhrp2/3 deletions and HRP2-based false-negative results in southern Ethiopia. Malar J. 2024;23:108. PubMed https://doi.org/10.1186/s12936-024-04904-3

7. Alemayehu GS, Blackburn K, Lopez K, Cambel Dieng C, Lo E, Janies D, et al. Detection of high prevalence of *Plasmodium falciparum* histidine-rich protein 2/3 gene deletions in Assosa zone, Ethiopia: implication for malaria diagnosis. Malar J. 2021;20:109. PubMed https://doi.org/10.1186/s12936-021-03629-x

8. Feleke SM, Reichert EN, Mohammed H, Brhane BG, Mekete K, Mamo H, et al. *Plasmodium falciparum* is evolving to escape malaria rapid diagnostic tests in Ethiopia. Nat Microbiol. 2021;6:1289–99. PubMed https://doi.org/10.1038/s41564-021-00962-4

9. Rogier E, McCaffery JN, Nace D, Svigel SS, Assefa A, Hwang J, et al. *Plasmodium falciparum pfhrp2* and *pfhrp3* gene deletions from persons with symptomatic malaria infection in Ethiopia, Kenya, Madagascar, and Rwanda. Emerg Infect Dis. 2022;28:608–16. PubMed https://doi.org/10.3201/eid2803.211499

10. Vera-Arias CA, Holzschuh A, Oduma CO, Badu K, Abdul-Hakim M, Yukich J, et al. High-throughput *Plasmodium falciparum hrp2* and *hrp3* gene deletion typing by digital PCR to monitor malaria rapid diagnostic test efficacy. eLife. 2022;11:e72083. PubMed https://doi.org/10.7554/eLife.72083

11. Leonard CM, Assefa A, McCaffery JN, Herman C, Plucinski M, Sime H, et al. Investigation of *Plasmodium falciparum* pfhrp2 and pfhrp3 gene deletions and performance of a rapid diagnostic

test for identifying asymptomatic malaria infection in northern Ethiopia, 2015. Malar J. 2022;21:70. PubMed https://doi.org/10.1186/s12936-022-04097-7

12. Golassa L, Messele A, Amambua-Ngwa A, Swedberg G. High prevalence and extended deletions in *Plasmodium falciparum* hrp2/3 genomic loci in Ethiopia. PLoS One. 2020;15:e0241807. PubMed https://doi.org/10.1371/journal.pone.0241807.

13. Gamboa D, Ho MF, Bendezu J, Torres K, Chiodini PL, Barnwell JW, et al. A large proportion of *P. falciparum* isolates in the Amazon region of Peru lack *pfhrp2* and *pfhrp3*: implications for malaria rapid diagnostic tests. PLoS One. 2010;5:e8091. PubMed https://doi.org/10.1371/journal.pone.0008091

14. Baker J, McCarthy J, Gatton M, Kyle DE, Belizario V, Luchavez J, et al. Genetic diversity of *Plasmodium falciparum* histidine-rich protein 2 (PfHRP2) and its effect on the performance of PfHRP2-based rapid diagnostic tests. J Infect Dis. 2005;192:870–7. PubMed https://doi.org/10.1086/432010

Appendix Table 1. Reassessing *pfhrp3*-deleted samples*

		Primer to 5′ end exon of 2 (4plex qPCR) qPCR pair 1	
Primers		*pfhrp2*$^+$/*pfhrp3*$^-$	*pfhrp2*$^-$/*pfhrp3*$^-$
Primers to 3′ end of exon 2 (modified 4plex qPCR) qPCR pair 2	*pfhrp2*$^-$/*pfhrp3*$^+$	0	2
	pfhrp2$^+$/*pfhrp3*$^-$	14	0
	pfhrp2$^-$/*pfhrp3*$^-$	0	0
	pfhrp2$^+$/*pfhrp3*$^+$	5	0
Total		19†	2

*Samples with a *pfhrp3* deletion as detected by 4plex qPCR with *pfhrp3* primers binding to the 5′ end of *pfhrp3* exon 2 (qPCR pair 1 [*3*]; this is the original published 4plex qPCR [3]) were reassessed by a modified 4plex qPCR with *pfhrp3* oligonucleotides targeting the 3′ end of *pfhrp3* exon 2 (qPCR pair 2 [*4*]**)**.
†Two samples not reassessed because of insufficient template DNA.

Appendix Table 2. Profiling the *pfhrp3* gene locus*

Sample type	Sample ID	qPCR pair 1	qPCR pair 2	PCR pair 3	PCR pair 4	PCR pair 5
Positive controls	3D7	+	+	+	+	+
	Sample 1	+	+	–	+	+
	Sample 2	+	+	+	+	+
Negative controls	HB3	–	–	–	–	–
	Sample 3	–	–	–	–	–
	Sample 4	–	–	–	–	–
Discordant samples	Sample 5	–	+	–	–	–
	Sample 6	–	+	–	–	–
	Sample 7	–	+	–	–	–
	Sample 8	–	+	–	–	–
	Sample 9	–	+	–	–	–

*This table displays the pfhrp3 profiles for various samples as analyzed by 5 distinct PCR assays. Each column represents the results from a different assay. The 5 samples (samples 5–9) negative for *pfhrp3* according to 4plex qPCR pair 1 (*3*) (this is the original published 4plex qPCR [*3*]) but positive for *pfhrp3* according to qPCR pair 2 (*4*) (Appendix Table 1) were reassessed with further commonly used PCR assays (primer pairs 3–5). Controls are *P. falciparum* laboratory strains and samples from the Ethiopian cohort that were either 4plex qPCR *pfhrp3* positive (samples 1 and 2) or negative (samples 3 and 4). qPCR pair 1, 4plex qPCR: 5′ end exon 2; qPCR pair 2, 4plex qPCR: 3′ end exon 2; +, amplicon detected at expected bp length; –, no amplification.

Appendix Table 3. Summary of previous studies on *pfhrp2*/*3* deletions in Ethiopia*

Author	Publication year	Year of sample collection	Study area in Ethiopia	Method	*pfhrp2*⁻/*pfhrp3*⁻ double deletion	*pfhrp2*⁻ single deletion†	*pfhrp3*⁻ single deletion	No. of tested samples
Kamaliddin et al. (*5*)	2024	2017–2018 and 2020–2022	Countrywide (7 of the 9 national regional states)	Single-step PCR and droplet digital PCR	17.2	21.9	69.1	233
Mekonen et al. (*6*)	2024	2021	South and southwestern	Nested PCR	13.2	27.3	30.5	249
Alemayehu et al. (*7*)	2021	2018	Northwest (Benishangul-Gumuz)	Single-step PCR	5.8‡	17.9	9.2	218
Feleke et al. (*8*)	2021	2017–2018	Northwest/north/southwest (Amhara/Tigray/Gambella)	Single-step PCR, molecular inversion probe deep sequencing and WGS	22	4.4	31	610
Rogier et al. (*9*)	2022	2017	North/south/central	Single-step PCR (*pfhrp2*) and nested PCR (*pfhrp3*)	15	10	20	20
Vera-Arias et al. (*10*)	2022	2016	Southwest (Jimma)	Droplet digital PCR	2.1	0	74.5	47
Leonard et al. (*11*)	2022	2015	North (Amhara/Afar/Tigray)	Single-step PCR (*pfhrp2*) and nested PCR (*pfhrp3*)	40	6.6	53.3	15
Golassa et al. (*12*)	2020	2015	Central (Adama)	Semi-nested PCR and DNA sequencing	100	0	0	50

*Deletions are in %. WGS, whole-genome sequencing.
†Data for exon 2 of the *pfhrp2* gene deletion.
‡Only a subset (n = 86, PfHRP2 RDT negatives) of total samples analyzed.

Appendix Table 4. Sequences of primers used for *pfhrp3* profiling

Pair	Primer name	Sequence 5′-3′	Binding site in *pfhrp3*	Reference
Pair 1	Fwd 1	CTCCGAATTTAACAATAACTTGTTTA	Exon 2: 5′ region	Krüger et al. (*3*)
	Rev 1	CAGCTACATGATGTGCATG	Exon 2: 5′ region	
	Probe	GAAAGTCAAGCACATGCAG	Exon 2: 5′ region	
Pair 2	Fwd 2	ACGGATTTCATTTTAACCCTTCACGA	Exon 2: 3′ region	Grignard et al. (*4*)
	Rev 2	TGAGAATCATCAAAACAAGCATTAGC	Exon 2: 3′ region	
	Probe	ACAATTCCCATACTTTACATCA	Exon 2: 3′ region	
Pair 3	Fwd 3	TATCCGCTGCCGTTTTTGCTTCC	Exon 1	Gamboa et al. (*13*)
	Rev 3	TGCATGATGGGCATCACCTG	Exon 2: 5′ region	
Pair 4	Fwd 3	TATCCGCTGCCGTTTTTGCTTCC	Exon 1	
	Rev 4*	TCGTGAAGGTTAAAATGAAATCCGT	Exon 2: 3′ region	Grignard et al. (*4*)
Pair 5	Fwd 2	ACGGATTTCATTTTAACCCTTCACGA	Exon 2: 3′ region	
	Rev 5	TGGTGTAAGTGATGCGTAGT	Exon 2: 3′ end	Baker et al. (*14*)

*Reverse complement of Fwd 2.

Appendix Table 5. PCR assays for *pfhrp3* profiling

Primer combination	Amplicon length, bp	PCR reaction mixture and thermal cycling conditions
Pair 1 (Fwd 1/Rev 1/Probe)	119	qPCR: 2× TaqMan Multiplex Master Mix; 0.4 μM primer; 0.15 μM probe<br>95°C 20 s (1×); 95°C 3 s, 62°C 150 s (45×); 40°C 30 s (1×)
Pair 2 (Fwd 2/Rev 2/Probe)	82	qPCR: 2× TaqMan Multiplex Master Mix; 0.4 μM primer; 0.15 μM probe<br>95°C 20 s (1×); 95°C 3 s, 62°C 150 s (45×); 40°C 30 s (1×)
Pair 3 (Fwd 3/Rev 3)	301	PCR: HotStarTaq DNA Polymerase (incl. 1.5 mM $MgCl_2$); 0.3 μM primer<br>95°C 15 min (1×); 94°C 30 s, 65°C 30 s, 72°C 60 s (40×); 72°C 5 min (1×); 4°C
Pair 4 (Fwd 3/Rev 4)	663	PCR: HotStarTaq DNA Polymerase (incl. 1.5 mM $MgCl_2$); 0.3 μM primer<br>95°C 15 min (1×); 94°C 30 s, 61°C 30 s, 72°C 60 s (40×); 72°C 5 min (1×); 4°C
Pair 5 (Fwd 2/Rev 5)	307	PCR: 1x AmpliTaq Gold Polymerase with Buffer II; 1.5 mM $MgCl_2$; 0.2 mM dNTPs; 0.2 μM primer<br>95°C 10 min (1×); 95°C 15 s, 55°C 30 s, 72°C 60 s (40×); 72°C 5 min (1×); 4°C

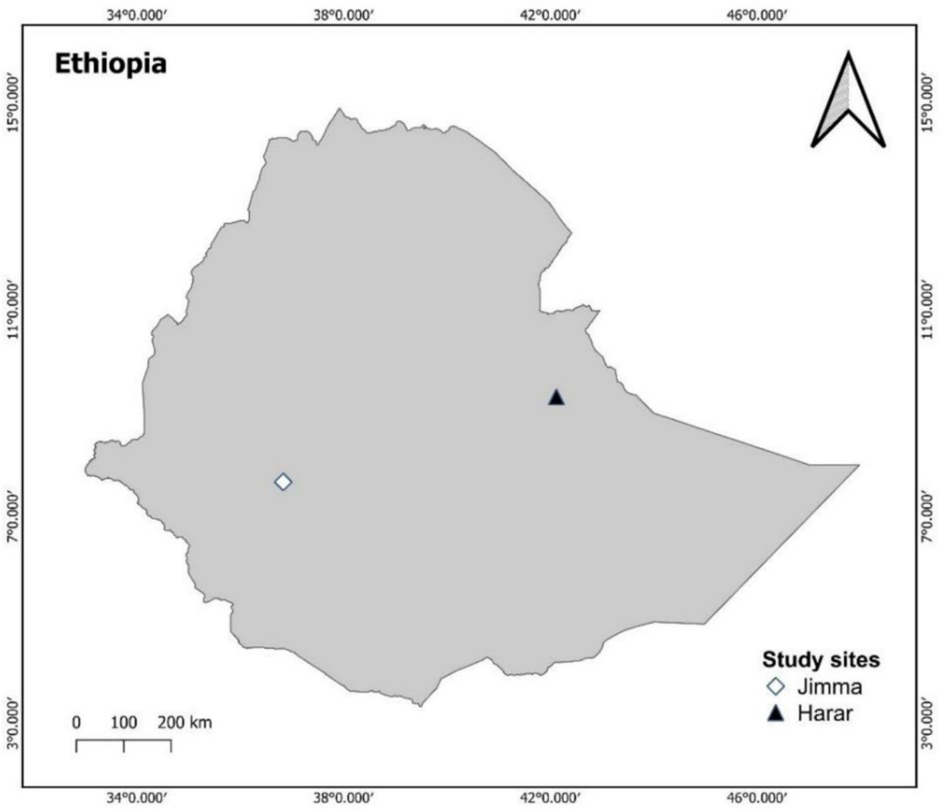

Appendix Figure 1. Study area (Jimma, Harar). The map shows the sampling regions and was generated using QGIS software (version 3.42.0).

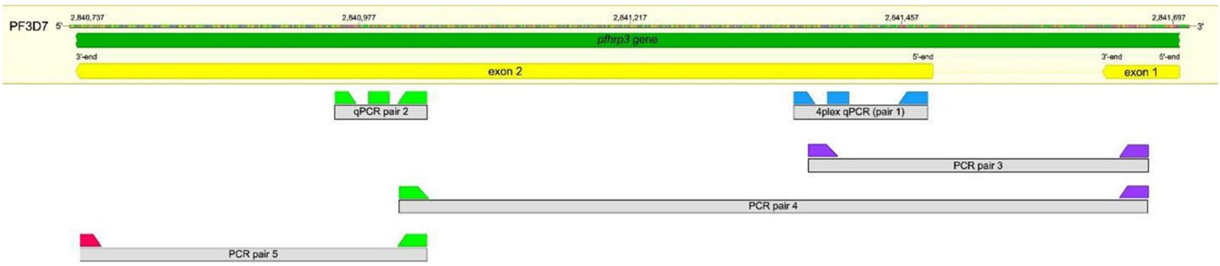

Appendix Figure 2. Schematic overview of *pfhrp3* primer binding regions. To profile the *pfhrp3* gene locus in samples that showed a *pfhrp3* gene deletion by 4plex qPCR, additional nucleic acid amplification assays were performed using different oligonucleotide sets. The figure shows the 3D7 reference genome (NCBI RefSeq accession no. NC_004331) and illustrates the binding region of the *pfhrp3*-specific oligonucleotide sets used. Oligonucleotide set 1 (4plex qPCR pair 1 [*3*]; this is the original published 4plex qPCR [*3*]) and oligonucleotide set 2 (qPCR pair 2 [*4*]) were run as 4plex qPCR assays, primer pairs 3–5 as conventional, singleplex PCR assays and analyzed by capillary gel electrophoresis. The figure was created using Geneious Prime (version 2023.2.1) and adapted accordingly. Sequences of primers used for *pfhrp3* profiling are shown in Appendix Table 4.
